# Climate Control on Tree Growth at the Upper and Lower Treelines: A Case Study in the Qilian Mountains, Tibetan Plateau

**DOI:** 10.1371/journal.pone.0069065

**Published:** 2013-07-11

**Authors:** Bao Yang, Minhui He, Thomas M. Melvin, Yan Zhao, Keith R. Briffa

**Affiliations:** 1 Key Laboratory of Desert and Desertification, Cold and Arid Regions Environmental and Engineering Research Institute, Chinese Academy of Sciences, Lanzhou, China; 2 Climatic Research Unit, School of Environmental Sciences, University of East Anglia, Norwich, United Kingdom; 3 Institute of Geographic Science and Natural Resources Research, Chinese Academy of Sciences, Beijing, China; The Pennsylvania State University, United States of America

## Abstract

It is generally hypothesized that tree growth at the upper treeline is normally controlled by temperature while that at the lower treeline is precipitation limited. However, uniform patterns of inter-annual ring-width variations along altitudinal gradients are also observed in some situations. How changing elevation influences tree growth in the cold and arid Qilian Mountains, on the northeastern Tibetan Plateau, is of considerable interest because of the sensitivity of the region’s local climate to different atmospheric circulation patterns. Here, a network of four Qilian juniper (*Sabina przewalskii* Kom.) ring-width chronologies was developed from trees distributed on a typical mountain slope at elevations ranging from 3000 to 3520 m above sea level (a.s.l.). The statistical characteristics of the four tree-ring chronologies show no significant correlation with increasing elevation. All the sampled tree growth was controlled by a common climatic signal (local precipitation) across the investigated altitudinal gradient (520 m). During the common reliable period, covering the past 450 years, the four chronologies have exhibited coherent growth patterns in both the high- and low-frequency domains. These results contradict the notion of contrasting climate growth controls at higher and lower elevations, and specifically the assumption that inter-annual tree-growth variability is controlled by temperature at the upper treeline. It should be stressed that these results relate to the relatively arid conditions at the sampling sites in the Qilian Mountains.

## Introduction

Owing to its high resolution and reliable dating, dendrochronology plays an important role in studies of past climate change [1], [2]. Recently, tree-ring chronologies have been used to reconstruct past climate history over a large range of spatial and temporal scales [3]–[5]. These extended records help us to better understand climate change in a longer-term context. In dendroclimatological studies, it is generally observed that relationships between tree growth and recorded climate parameters can be species- and context-dependent, since the impacts of environmental factors on tree growth may vary along altitudinal, latitudinal and longitudinal gradients [6]–[9]. On the basis of physiological associations between plant growth and climate controls at high elevation [10], we might expect that low temperatures limit tree growth at the upper treeline [11]. For example, previous studies have reported the general “principle of tree growth-climate relationships”, whereby tree growth at the upper treeline is controlled by temperature while that at the lower treeline is controlled by precipitation [12]–[14]. A considerable body of research in many regions has generally supported this principle of tree growth-climate relationships [15]–[18]. However, in contrast, uniform growth patterns (where the tree growth is controlled by temperature at both tree lines or precipitation at both tree lines) have also been observed in the arid regions of western central Asia [19], in the semi-humid region of the southern Tibetan Plateau [20], in the semi-humid climates of the southeastern [21] and eastern flanks [22] of the Tibetan Plateau, and in the monsoonal rainfall patterns of northwestern Argentina [23]. Thus, clarifying the association between geography, elevation and climate influences on tree growth still requires further research.

In the past decade, much tree-ring focused research has been conducted on the northern [24]–[26] and southern Tibetan Plateau (TP) [27]–[29], due to the region’s high sensitivity to climate change [30]. Specifically, research in the Qilian Mountains region, on the northern margin of the TP, is both challenging and of great importance as its location makes it sensitive to the varying influences of different atmospheric circulation patterns [31]. Despite the many studies focusing on dendroclimatic research in this mountainous region, results have been inconsistent: some have suggested that the tree-ring widths are predominantly influenced by temperature variation [32]–[34], whereas others have indicated a significant effect of local precipitation [31], [35], [36]. Meanwhile, Tian et al. [37] demonstrated that both temperature and precipitation had significant effects on tree growth, concluding that both should be considered in climate reconstruction. Consequently, it is still necessary to undertake additional, localized studies to further explore the complexity of tree growth-climate relationships in what is a cold and arid region.

In this study, we used dendrochronological methods to investigate the effects of climatic variability on the local growth of Qilian juniper (*Sabina przewalskii* Kom.) in the elevation range 3000–3520 m a.s.l. in the cold and arid Qilian Mountains, northeastern TP. The objectives of the study were first to test whether altitude has significant influence on local tree growth patterns sampled along a typical mountain slope; second to identify the climatic factor principally responsible for these growth patterns, and third to assess whether local tree growth patterns have remained consistent for the past few hundreds of years.

## Materials and Methods

All necessary permits were obtained for the described field studies from the Administration of Gansu Qilianshan National Nature Reserve.

### Study Area and Climate

The Qilian Mountains are located at the northern margin of the TP, which is presently characterized by a continental arid climate and primarily influenced by the westerlies [31], [37]. Winters in the Qilian Mountains are quite cold and dry, while summers are relatively hot. The environment of the Qilian Mountains becomes increasingly arid from east to west.

Our study area lies in the western distribution limit of Qilian juniper in the Qilian Mountains (Fig. S1), located in the arid interior of northwest China. The closest available meteorological station is Jiuquan (39°46′ N, 98°29′ E, 1477 m a.s.l.). This is located some 1500 m below the elevation of the study sites. To evaluate the suitability of our selected climate data, we compared daily temperature and precipitation variations along an altitudinal gradient of ∼700 m using observations collected at an automated weather station, which was installed very close to our sampling site and belonged to the same climate type as our present study region in the central Qilian Mountains. Consistent variations in temperature and precipitation were found along the altitudinal gradient (Table S1). The daily average temperatures were −0.53°C, 0.10°C and 1.95°C at the upper, middle and lower sites, respectively, indicating a consistent lapse rate with elevation. For the monitoring period 2011 and 2012, the average annual precipitation was 487 mm, 490 mm and 453 mm at the upper limit site, middle limit site and lower limit site; this demonstrates that annual precipitation does not significantly change with elevation. Further, we compared our selected Jiuquan station data with the gridded monthly dataset from the Climatic Research Unit (CRU) TS 3.1. The method of interpolation used for producing TS 3.1 is regarded as state-of-the-art [38]. It was found that our selected Jiuquan station data are significantly correlated with monthly mean temperature and precipitation from the CRU TS 3.1 dataset (Table S2), which were derived from two grid points (39.25°N, 98.25°E and 39.25°N, 98.75°E) with respective elevations of 3783 m and 3237 m (similar to our sampling elevations) over the common period 1951–2009. The high degree of similarity demonstrates a rather homogenous climate over the study area. Therefore, we are confident that the Jiuquan station has good regional representation and can be used to reflect general climatic conditions at our sampled sites. The observed mean annual temperature (1951–2011) for Jiuquan is 7.48°C, with mean temperatures in January (the coldest month) and July (the warmest month) of −9.43°C and 21.98°C, respectively. The mean annual minimum temperature is 1.07°C and the mean annual maximum temperature is 14.90°C. The mean annual precipitation is only about 87 mm, of which 10.8% falls during October to February, and 79% is concentrated in May–September and coincident with the highest temperatures (Fig. S2). The winter (December–February) and summer (May–September) relative humidity is 53.19% and 46.63%, respectively. Strong winds are frequent throughout the year.

### Tree-ring Sampling and Chronology Development

Qilian juniper (*Sabina przewalskii* Kom.) trees were sampled in open stands on a south-facing slope. Core samples were extracted at breast height using increment borers and 2–3 cores (5.15 mm diameter) were collected, each from a different direction within each tree. Trees were sampled at different discrete sites along an altitudinal range of 3000–3520 m a.s.l. on a typical mountain slope (see Fig. S3). Herein, the term “typical” means that the selected tree-ring cores were collected from sites with an arid climate representative of conditions in the Qilian Mountains, northern TP. In total, 90 cores from 46 trees were sampled at the “higher-altitude” forest site (>3400 m); 111 cores from 47 trees were sampled at the “higher-mid altitude” forest site (3340–3400 m); 110 cores from 52 trees were sampled at the “mid-altitude” forest site (3220–3340 m); and 109 cores from 50 trees were sampled at the “lower-altitude” forest site (<3220 m). The median altitudes of the four elevation sites were 3465 m, 3372 m, 3293.5 m and 3149 m, respectively. Considering each series’ elevation, we calculated weighted mean site elevations: 3462 m, 3373 m, 3293 m and 3151 m for the four sites.

In the laboratory, the sampled increment cores were prepared following standard dendrochronological techniques [39]. Ring widths were measured with a LINTAB measuring system at a resolution of 0.01 mm, and all cores were cross-dated by visual inspection [40] and by statistical tests (sign-test and t-test) using the software package TSAP-Win [41]. The quality of the ring-width crossdating was further checked using the specialized software package COFECHA [42]. These methods ensured the exact dating for each annual ring-width series.

Ring-width chronologies for each of the four sites were developed using the ARSTAN software [43]. To reduce the potential heteroscedasticity commonly found in the raw ring-width measurements, a data-adaptive power transformation was firstly applied [44]. We fitted most of the ring-width measurement series with either negative exponential curves or straight lines of any slope. If both of these curve types failed, a cubic smoothing spline with a 50% frequency response cutoff width equal to two thirds of the individual series length was applied. Tree-ring indices were calculated as the difference between the power-transformed ring-width measurements and the values of the fitted curves. This method avoids possible “end-effect” bias in the resulting tree-ring chronologies due to index inflation after calculating ratios [44]. The detrended tree-ring series were averaged to a standard site chronology using a biweight robust estimate of the mean to minimize the influence of outliers [39]. Owing to a changing sample size, the chronology variance was stabilized using sample size and an average correlation criterion based on the method described by Osborn et al. [45]. Finally, two versions of the chronology were produced: the residual (RES) and standard (STD) chronologies. Thereafter, the “pre-whitened” RES chronology was used for the analysis of tree growth-climate relationships where biologically related persistence has been removed. The STD chronology retains more low-frequency variability [43] and was used to represent the longer-term tree growth trends over the past 400 to 500 years. Further, several descriptive statistics were calculated for the standard chronologies [1]. The standard deviation (SD) estimates the variability of measurements for the whole series; the mean sensitivity (MS) quantifies the inter-annual variation in growth between two adjacent rings [1], and the first-order autocorrelation (AC1) assesses the persistence of the site chronologies. Common signal strength was evaluated by the percent variance explained by the first principal component (PC#1) and by the mean inter-series correlation (Rbar). The signal-to-noise ratio (SNR) serves as an expression of the strength of the observed common signal among trees. The expressed population signal (EPS) [46] is used to determine the statistically reliable time periods of the chronologies using a 30-year moving window with 15-year overlaps. The approximate threshold for an acceptable EPS was considered by Wigley et al. [46] to be 0.85. However, we note that the application of the Rbar- and sample number-based EPS threshold used here relates principally to the high-frequency variability of the chronology, and has less relevance to the assessment of long-timescale (e.g. centennial and longer) variability of the chronologies [47]. Nevertheless, the EPS threshold of 0.85 still provides a useful and directly comparable indication of chronology reliability across sites. The formulae for calculating the EPS and SNR can be found in Wigley et al. [46].

### Tree Growth-climate Relationship Analysis

Monthly values of mean temperature, mean maximum temperature, mean minimum temperature, precipitation and relative humidity from Jiuquan meteorological station are available for the common period 1951–2011. In addition, the Palmer Drought Severity Index (PDSI) [48] was also used to investigate the influence of drought stress on tree growth. The PDSI data used here were taken from the global analysis published by Dai et al. [49]. Data for the 0.5°×0.5° grid box closest to our sampling sites (38.75°N, 98.75°E) were extracted for the period 1951–2005. We performed correlation and “response function” analysis in which tree-ring chronologies and various monthly mean series of climate data were compared. The DENDROCLIM2002 software [50] was implemented using a 15-month window (including climate data) from prior July to current September where “current” indicates a month coincident with the year in which tree growth occurred. To examine the integrated impact of precipitation and temperature on radial growth at the four different elevation sites, response surface regressions were conducted using STATISTICA (version 7.0; StatSoft Inc., Tulsa, USA). Various seasonal means of the climatic variables and their correlations with PC#1 were presented to allow an assessment of the common pattern of climate influence on tree growth at the study area. Finally, partial correlations were also calculated to determine the specific climatic factor most strongly associated with local tree growth patterns.

Furthermore, to assess whether the identified tree growth-climate relationships were consistent for different tree-ring ages, we also classified all the sampled trees into two sub-groups: an older tree group (>300 years old, 56 trees/103 cores) and younger tree group (≤300 years old, 139 trees/317 cores). The age-dependent tree-ring chronologies were subjected to the same correlation and response function analysis as that used to investigate the climate influence on the four altitudinal chronologies.

### Assessment of the Consistency in Tree Growth Patterns for the Past Hundreds of Years

To distinguish the variation in patterns between different frequency domains, we performed high-pass and low-pass filtering on the raw standard tree-ring chronologies over their common reliable period. Correlation calculations with the original unfiltered data, the high-pass filtered data and the low-pass filtered data were performed. Since there are strong autocorrelations in the low-pass filtered data, the degrees of freedom for significance testing was adjusted following the method described by Bretherton et al. [51]. Furthermore, the multi-taper Method (MTM) of spectral analysis [52] was applied to compare the high- and low-frequency variabilities of the four tree-ring chronologies over their “most reliable” period 1560–2011. In addition, the four chronologies were analyzed using principal components analysis (PCA). The first principal component (PC#1) represents the pattern of chronology variance that is common across all sites and is an indicator of the optimum “regional” tree growth signal.

## Results

### Statistical Characteristics of Standard Tree-ring Chronologies

**Figure 1:**
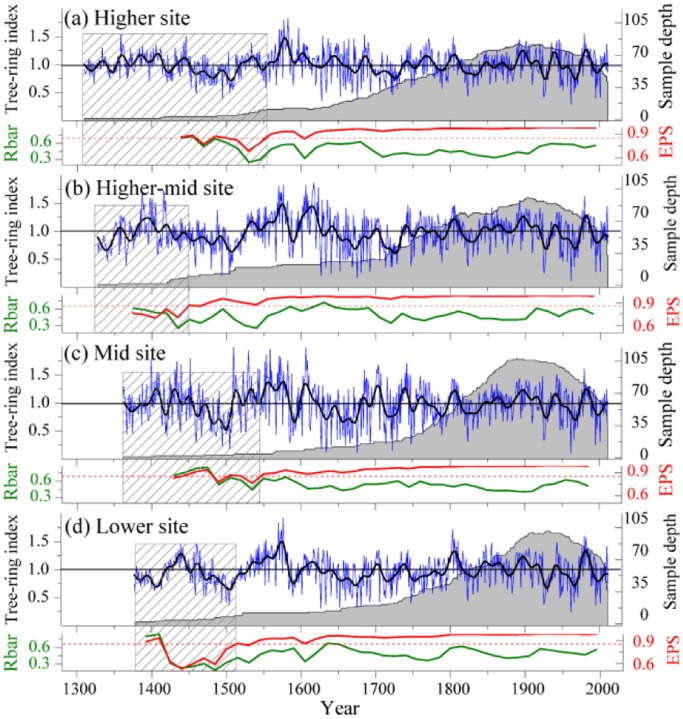
Comparisons of the four altitudinal standard chronologies. a) The higher site chronology; b) the higher-mid site chronology; c) the mid site chronology; d) the lower site chronology. The blue lines are the original series and the black lines are the corresponding 11-year Fast Fourier Transformed (FFT) series with the corresponding sample depth (gray area), expressed population signal (EPS, red lines) and Rbar (olive lines) based on a 30-year window with 15-year overlaps. The dashed red line shows the EPS = 0.85 cut-off. The rectangles with gray oblique lines indicate the less reliable periods for the individual standard chronologies.

The statistical characteristics of the four chronologies, each representing a different elevation, are presented in Table 1. The highest site yielded the longest time span, followed by the higher-mid site. The mean segment lengths (MSL) of the four series ranged from 208 years (mid site) to 249 years (higher site). The lowest site had the highest average growth rate (AGR), while the other three higher sites were similar to each other; the lowest growth rate was observed at the higher-mid sampling site. The number of “missing” rings was highest at the lower sampling site. Specifically, missing ring rates for the lowest growth in 1962 (and 1676), from the low to high elevation sites respectively, were 13.33% (28.57%), 10.94% (50%), 6.15% (28.12%) and 6.78% (16.67%). Standard deviation (SD) and mean sensitivity (MS) reached their highest/lowest values at the mid/higher sampling sites. The values of both SD and MS at the different sites were not correlated with site elevation. During the common period 1850–1950, the first order autocorrelations (AC1) of the four chronologies ranged from 0.22 (higher site) to 0.33 (lower site). The highest loadings on PC#1, and the highest peak EPS and SNR values all occurred at the mid-altitude site, while the lowest values all occurred at the lower elevation site. To achieve an EPS >0.85, 5–6 trees were required in all of the four chronologies. Statistics relating to the standard chronologies (sample depth, Rbar and EPS over the full chronology periods) at the four sampling sites are given in Fig. 1. All the chronologies are considered reliable (on the basis of EPS) for the period starting from AD 1560. The relevant data will be deposited in the NOAA National Climatic Data Center (www.ncdc.noaa.gov).

**Table 1:** General characteristics of the four “standard” tree-ring chronologies.

251659264	Lower site	Mid site	Higher-mid site	Higher site
Time span	1378–2011	1362–2011	1329–2011	1310–2011
Trees/Cores	50/109	52/110	47/111	46/90
MSL	211	208	247	249
AGR	0.61	0.40	0.39	0.43
MRR	1.21%	1.11%	1.13%	0.55%
SD	0.26	0.34	0.31	0.24
MS	0.26	0.37	0.31	0.25
AC1	0.33	0.25	0.31	0.22
PC#1	29.10	39.81	36.49	34.07
SNR	61.04	79.73	74.73	70.18
EPS	0.98	0.99	0.99	0.99
EPS >0.85	1530/5	1545/6	1450/6	1560/6
R1	0.54	0.52	0.53	0.53
R2	0.66	0.62	0.59	0.78
R3	0.53	0.51	0.52	0.52

MSL is the mean segment length; AGR is the average growth rate; MRR is the missing-ring rate; SD is the standard deviation; MS is the mean sensitivity; AC1 is the first order autocorrelation; PC#1 is the variance explained by the first principal component; SNR is the signal-to-noise ratio; EPS is the expressed population signal; EPS >0.85 indicates the year and number of trees with EPS exceeding 0.85; R1 is the all-series Rbar; R2 is the within-trees Rbar; R3 is the among-trees Rbar.

### Correlation and “Response Function” Analysis

**Figure 2:**
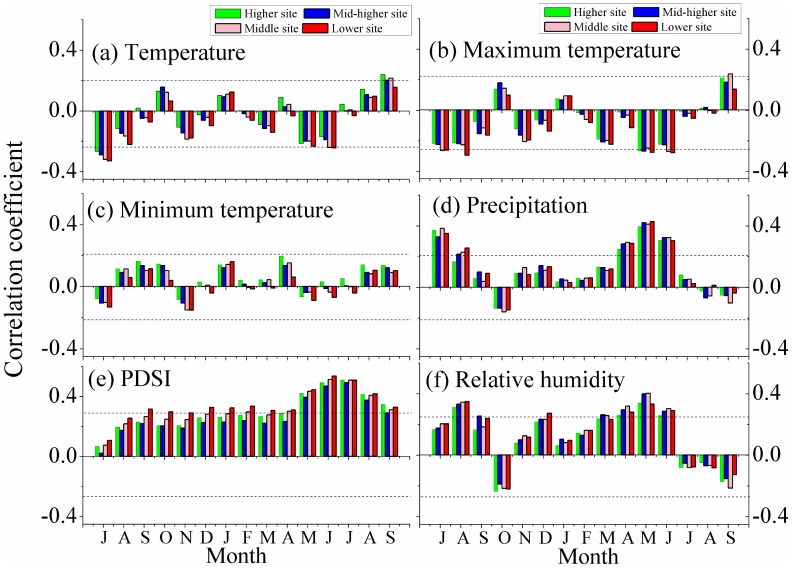
Correlations between climate data and the four residual chronologies over their common periods. a) correlations with monthly temperature; b) correlations with monthly minimum temperature; c) correlations with monthly maximum temperature; d) correlations with monthly precipitation; e) correlations with monthly PDSI; f) correlations with monthly relative humidity. The horizontal dashed lines indicate the p = 0.05 significant level.

**Figure 3:**
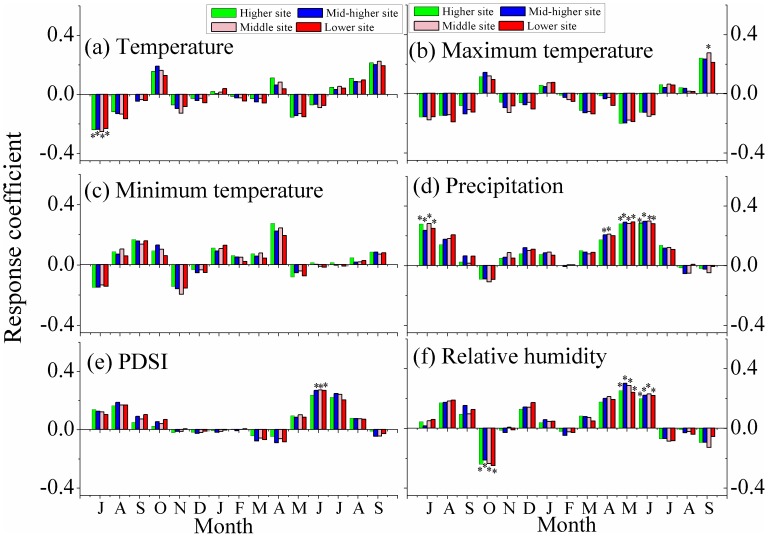
Response function results between climate data and the four chronologies over their common periods. The detailed panels (a), (b), (c), (d), (e) and (f) follow the order in Figure 2. Response functions significant at the 0.05 level are marked with an asterisk.

The general patterns of statistical associations between inter-annual tree growth and climate variables are illustrated in Figs. 2 and 3. The strengths of negative correlations with previous July mean temperature (Fig. 2a) appear to decrease with increasing elevation: the correlation coefficients range from −0.27 to −0.33. In the response function analysis (Fig. 3a), the association with mean temperature in prior July is significant at the p<0.05 level at all sites but does not reach the p<0.01 level, even at the lowest site. Therefore, the influence of previous July temperature is not significantly affected by elevation. For the remaining months, none of the correlations with mean temperatures are significant at the p<0.05 level and no effect of elevation on these associations can be identified. The highest negative correlations between tree-ring series and maximum temperature (Fig. 2b) occurred in the current May at both the higher (−0.27) and higher-mid (−0.27) sampling sites. At the mid (−0.27) and lower (−0.28) sites the most negative associations were seen in June. However, the corresponding response functions (Fig. 3b) indicated current May and June associations with tree growth were not significant (p<0.05) at any of the four altitudinal sites. The influence of monthly minimum temperature (Figs. 2c, 3c) on tree growth at the four sites is not significant and no elevation trends are identified.

Compared to temperature, precipitation or precipitation-induced water variability had greater apparent influences on local tree growth. From Figs. 2d and 3d, significant (p<0.05) correlations with precipitation are apparent in the previous July and current May, as well as in current June, over the complete altitudinal range. Correlation coefficients were also significant in current April at all four sites, but the associated response functions indicate that significant (p<0.05) associations occurred in the higher-mid and mid sites. The major feature of the PDSI series (Fig. 2e) is the consistently significant associations apparent in the previous September to current September at the lowest sampling site and also during the growing season (May to September) at the other three higher sites. However, response function analysis (Fig. 3e) indicated that only the PDSI in current June significantly (p<0.05) influenced tree growth at the higher-mid (r = 0.27), mid (r = 0.27) and lower (r = 0.27) sites. Correlation coefficients (Fig. 2f) indicated that increased previous August relative humidity had a positive influence on tree growth at all of the four sites. The same is true for the current March-June at the mid and higher-mid sites and for current April–June at the higher and lower altitudinal sites. Response function analysis of relative humidity (Fig. 3f) presented more coherent trends, with significant (at the p<0.05 level) influences of humidity during the previous October, current May and June apparent at all tree-ring sites. There is no evidence for the influence of relative humidity on local tree-ring growth at any other sites in any other months.

Correlation and response functions calculated between age-dependent chronologies and climate data are presented in Fig. S4. No significant (p<0.05) discernible differences in the growth-climate relationships can be identified, and all featured significant negative correlation and response functions with current May maximum temperature and previous October relative humidity. Meanwhile, there are significant positive correlation and response functions for previous July as well as current May–June precipitation, current June PDSI and current May relative humidity.

### The Driving Climatic Factors for Local Tree Growth

The calculated correlations between the four residual chronologies and seasonally aggregated climate data are presented in Table 2. The results indicate that only a weak negative influence of winter temperatures (from the previous November to the current February) on tree growth can be detected. Current May–June temperatures correlated negatively with the four tree-ring series, especially for the maximum temperature. Increased precipitation and/or water supply (positive PDSI or relative humidity) in May–June enhanced local tree growth. On the annual scale, simple averaging of the previous July to current June precipitation yielded the highest correlations with the four series, further confirmed by the correlations with the high-frequency (the corresponding first-order difference series) and low-frequency (the 5-year smoothed series) variability of the RES chronology-based PC#1 time series.

**Table 2:** Correlation coefficients between the seasonally aggregated climate data and the four residual chronologies, the RES-chronology based PC#1 series, the corresponding first-order difference series of PC#1, and the 5-year smoothed series of PC#1 over their common periods (Meteorological data cover 1951–2011; PDSI covers 1951–2005).

	HS	HMS	MS	LS	PC#1	1st differenceseries	5-year smoothed series
p_11_-c_2_Tem	−0.02	−0.06	−0.07	−0.10	−0.06	−0.16	0.08
p_11_-c_2_ Tmax	−0.06	−0.10	−0.11	−0.15	−0.11	−0.21	0.04
p_11_-c_2_ Tmin	0.05	0.01	−0.00	−0.02	0.01	−0.10	0.16
c_5–6_ Tem	−0.25	−0.25	−0.29*	−0.30*	−0.27*	−0.32*	−0.16
c_5–6_ Tmax	−0.33*	−0.32*	−0.34**	−0.35**	−0.34**	−0.43**	−0.13
c_5–6_ Tmin	−0.06	−0.06	−0.07	−0.12	−0.08	0.03	−0.07
c_5–6_ Pre	0.48**	0.51**	0.51**	0.51**	0.51**	0.52**	0.43
c_5–6_ PDSI	0.46**	0.44**	0.49**	0.50**	0.48**	0.60**	0.36
p_7_-c_6_ Pre	0.56**	0.59**	0.60**	0.61**	0.60**	0.63**	0.58*
p_6_-c_5_ Pre	0.52**	0.54**	0.55**	0.58**	0.56**	0.59**	0.55*
p_8_-c_7_ PDSI	0.33*	0.30*	0.35**	0.39**	0.35**	0.40**	0.49
p_7_-c_6_ PDSI	0.28*	0.25	0.31*	0.35*	0.30*	0.33*	0.49
p_7_-c_6_ RH	0.30*	0.36**	0.36**	0.36**	0.35**	0.57**	−0.16

Tem = mean temperature; Tmax = maximum temperature; Tmin = minimum temperature; Pre = precipitation; PDSI = Palmer Drought Severity Index; RH = relative humidity; p means previous year; c means current year; HS means the higher-site; HMS means the higher-mid site; MS means mid-site; LS means lower-site. ** means correlation is significant at the p = 0.01 level, * means correlation is significant at the p = 0.05 level. The significance testing of the 5-year smoothed series was based on the adjusted degree of freedom method described by Bretherton et al. [51].

**Figure 4:**
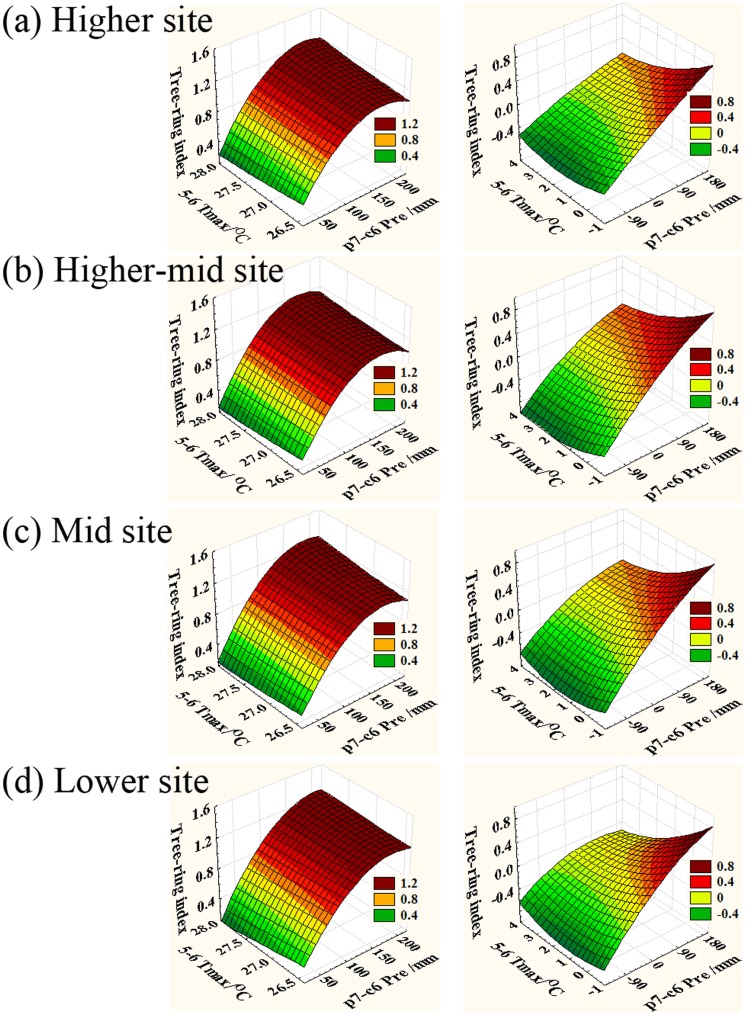
Results of the response surface regression analysis. The left panel is for the RES chronologies and the right panel is for the corresponding first-order difference series of the four elevation sites. a) The higher site chronology; b) the higher-mid site chronology; c) the mid site chronology; d) the lower site chronology. The X, Y and Z axes denote annual (previous July to current June) precipitation series, current May–June maximum temperature and residual tree-ring series, respectively.

Results from the response surface regression analysis, which was conducted between the four residual chronologies and instrumental climate data, are presented in Fig. 4. At the four elevation sites, wider tree-rings were formed if the annual precipitation at the Jiuquan reference station exceeded 150 mm, no matter how high or low the maximum temperature (left panel). Results from the first-order difference analysis (right panel) show that the associations between tree-ring series and precipitation may be much stronger if the maximum temperature is lower. Overall, Fig. 4 provides evidence that the associations between precipitation and tree-ring series are stronger, while the influences of temperature on tree growth are weaker at the four sites. These results are validated by the partial correlation analysis, since the only significant correlation is with the annual precipitation from previous July to current June (where significance is defined at the p<0.001 level). Hence, the annual precipitation is a major, significant climatic factor, and the one apparently most responsible for controlling local tree growth patterns.

### Variability of the Four Chronologies Over the Common “Most Reliable” Period AD 1560–2011

During the past “most reliable” 450 years, analysis of the high-frequency variability inferred from the four residual chronologies indicates that relatively narrow tree rings were produced in the same “pointer” years, regardless of elevation: for example, in the years 1634, 1676, 1748, 1926, 1962 and 1995. However, the variability of the ring width in the same “pointer” year shows no significant correlation with increased elevation. Taking the year 1962 as an example, the tree-ring widths were 0.32, 0.25, 0.18 and 0.37 from the high to low elevation sites, respectively. The results of correlation analysis, for the original unfiltered data (Table S3) and the high-pass filtered as well as low-pass filtered datasets (Table S4) over the common interval 1560–2011, demonstrate that all the chronologies were positively and significantly correlated with each other (p = 0.01). Furthermore, The MTM result (Fig. S5) shows that all the four series have the same significant (p = 0.05 level) high-frequency (∼2-year cycles) and low-frequency (∼200-year cycles and ∼40-year cycles) variability. There is no evidence that any of the four chronologies have any other significant cycles.

PCA over the period 1560–2011 demonstrated that only the first eigenvalue was greater than 1, and PC#1 accounted for 92.56% (89.53%) of the common variability extracted from the four residual (standard) chronologies. The corresponding PC#1 residual (standard) loadings were 0.96 (0.95), 0.96 (0.95), 0.98 (0.97) and 0.95 (0.92) for the higher site, higher-mid site, mid site and lower site, respectively.

**Figure 5:**
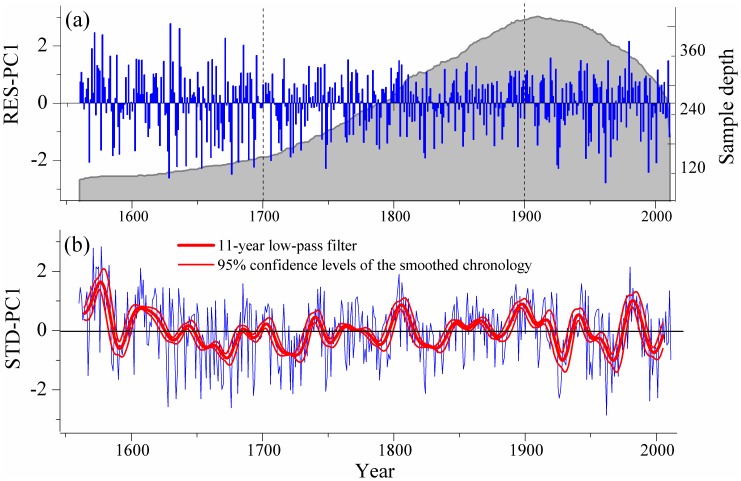
The PC#1 series and sample depth over the common reliable period 1560–2011. a) PC#1 series extracted from the four residual chronologies; b) PC#1 series (blue curve) extracted from the four standard chronologies and the corresponding 11-year low-pass filtered series (red heavy curve) with the 95% confidence levels (red thin curve).

Based on the above results and comparisons, we further analyzed the PC#1 time series for both the four residual (RES, Fig. 5a) and standard (STD, Fig. 5b) chronologies to detect local high- and low-frequency tree growth variability at longer time scales during the common reliable period of AD 1560–2011. Using the RES-based PC#1 series, the standard deviations (variation ranges) of the three periods 1560–1699, 1700–1899 and 1900–2011 were 1.18 (5.39), 0.86 (3.84) and 1.00 (4.95), respectively. Hence, there was greater high-frequency variability before 1700 and after 1900 than that during the 19^th^ and 20^th^ centuries. Based on the 11-year smoothed PC#1 series (Fig. 5b), we found that “favorable” climate periods extending continuously over more than ten years occurred in AD 1570–1591, 1603–1629, 1741–1751, 1801–1821, 1848–1880, 1891–1918, 1942–1951, 1980–1996; meanwhile, “detrimental” climate conditions primarily prevailed in AD 1592–1602, 1653–1687, 1712–1740, 1746–1758, 1782–1800, 1822–1847, 1881–1890, 1926–1941, 1952–1979, 1997–2011.

## Discussion

Based on the above rigorous statistical methods and results, we are confident that the statistical characteristics of the four chronologies have no correlation with elevation; altitude has no influence on the tree growth-climate associations; and annual precipitation from previous July to current June controls tree growth at both the upper and lower treelines. Further, the four chronologies have all maintained consistent high- and low-frequency variability over their “most reliable” period AD 1560–2011 in this cold and arid region of the northeastern Tibetan Plateau. However, the importance of temperature or precipitation in controlling tree growth with respect to altitude likely depends on the general climate of the area.

Here, the lack of influence of altitude on tree growth in our study region is consistent with previous research on the southern [20], [53], southeastern [21], [22] as well as northeastern [54] TP, where tree growth has shown consistent trends with elevation. For example, on the semi-humid southeastern TP (29.17–30.25°N, 93.20–95.58°E), Liang et al. [21] found that the radial growth of Smith fir showed a markedly similar response to common climatic signals, such as July minimum temperature, across a broad altitudinal range (3550–4390 m a.s.l.) in the Sygera Mountains. In the whole of western central Asia (35.17–40.17°N, 71.50–75.50°E), Esper et al. [19] investigated tree growth at 28 juniper sites in lower (about 2860 m a.s.l.) and higher (about 3440 m a.s.l.) elevation environments and showed similar ring-width variations at both elevations in the arid and high mountain environment. Further towards the northeastern region of the arid central Tianshan Mountains (43.75–43.98°N, 88.00–88.33°E), tree radial growth of *Picea schrenkiana* was similar over an altitudinal range of 1600–2700 m a.s.l. and was mainly controlled by the precipitation variability [55]. Still, in a low latitude humid region of North America (19.58°N, 103.62°W; mean annual precipitation 800–1000 mm, mean annual temperature ∼7°C), Biondi [56] found that precipitation was the main climatic signal apparent in a 400-year tree-ring chronology sampled at a high altitude site (3600–3700 m a.s.l.) in Nevado de Colima, Mexico. Thus, it is possible that the principle of temperature limitation at high elevation may not be applicable to the tropical treeline of North America [57], [58].

On the other hand, the universal interpretation of the above principle of limiting factors, which describes how tree growth is more sensitive to temperature at its upper distribution limit, has mostly been applied in the generally humid Alps [15], [17], [59], [60], [61]. In particular, Babst et al. [62] analyzed tree-ring width series from approximately 1000 sites, covering most of Europe and parts of North Africa (30–70°N/10° W-40°E), at elevations ranging from sea level to 2500 m a.s.l. in the Mediterranean mountain ranges. They found that temperature controlled forest productivity in high-elevation and high-latitude areas, whereas moisture-sensitive sites were widespread at low elevations in central and southern Europe. Such varied tree growth-climate relationships most probably arose in response to the regional variations in climate along the altitude gradients and geographical variability of sites investigated [63]. Similar tree growth variability along elevation gradients has also been found in the high-latitude continental boreal climate region of central and southern British Columbia [64], and also in the semi-arid western North America [65].

We now ask why there is no variation in the tree growth-climate relationship with altitude in our study region. We consider that the relatively arid conditions [31], [37] probably drive the consistent and coherent inter-annual variability of tree growth-climate relationships in this part of the Qilian Mountains. As observed from the correlation and response function results, the significant positive relationships with precipitation in late spring to early summer (April to June) and negative associations with May maximum temperature indicate that warm and dry conditions during the growing season hamper juniper growth at this arid site, whereas humid conditions favor tree growth, presumably due to increased soil moisture and reduced evaporative losses [1], [8]. Additionally, high temperatures coupled with low precipitation during prior late summers probably cause drought stress, stomata closure and reduced carbon assimilation [55]. High precipitation most likely enhances photosynthetic rates and the production of carbohydrates, which are stored in branches and needles over the winter [18], resulting in more stored food reserves for tree growth in the following year [14]. Meanwhile, the highest correlations observed between annual (previous July to current June) precipitation and the PC#1 time series indicate that local tree growth at the higher and lower sites is mainly driven by the annual precipitation from previous July to current June, which is consistent with previous studies in the vicinity of the study region [31], [66].

The consistent tree growth patterns for the past reliable 450 years yield more dendrochronological information for the study region. As inferred from Fig. 5a, local extreme climatic events may have caused the high frequency variability before the 18th century. On the other hand, the reduced sample depth (47–93 cores) of this period AD 1560–1699, compared to the other two periods (above 100 increment cores), may also be responsible for this observation. However, after the 20th century, the high sample depth demonstrates that the high frequency tree growth variability is likely to be the result of local climate variability. Because climatic extremes can have serious and damaging effects on human society and infrastructure as well as on ecosystems and wildlife, recent research has focused increasingly on extreme climatic events [67]–[70]. However, Easterling et al. [71] still stressed that one of the biggest problems in performing analyses of extreme climate events for most of the globe is a lack of high-quality, long-term data series. The long-term and high-resolution tree-ring records presented here can provide useful proxy information addressing this data gap. In addition, the low-frequency variability from the 11-year smoothed series is important for local climate change research, since this would be the foundation for sound regional climate modeling and for more reliable forecasts of trends in future precipitation variability in the cold and arid Qilian Mountains, similarly to equivalent studies in different regions [72]–[74].

## Conclusions

Four tree-ring width chronologies of *Sabina przewalskii* have been established. These have good sample depth and cover a typical mountain slope, ranging in altitude from 3000–3520 m a.s.l. in the cold and arid Qilian Mountains, on the northeastern Tibetan Plateau. Our results indicate that there is no significant influence of altitude on the statistical characteristics of the four tree-ring chronologies. In addition, the four chronologies present coherent inter-annual variability patterns not only over the instrumental period, but also during the past 450 years, as inferred from tree growth-climate relationships and correlation coefficients among the original unfiltered data, the high-pass filtered data and the low-pass filtered data for this period (1560–2011). Local tree growth is apparently controlled by the same climatic factor, regardless of elevation, tree age and frequency-domain: a result that may be explained by the relatively arid conditions of the study area. Moreover, the PC#1 series both from RES and STD chronologies yield additional information for future climate study in this cold and arid region.

After reviewing previous research using tree-rings to assess the effects of altitude, and in light of the data presented in this study, we conclude that a universal interpretation of the principle of limiting factors, which describes how tree growth is more sensitive to temperature at upper treeline environments, is not supported by data collected in the arid and semi-arid climate regions where water limitation is most important in tree growth systems. Additionally, the principle is not applicable to the semi-humid climate regions in most cases. Meanwhile, in the high-latitude regions of North America as well as in the generally humid central and southern Europe, the above generalized growth limiting factors can be detected. In other intermediate areas, we cannot recommend the generalisations based on the differing controls across the altitudinal gradient as hypothesized in the introduction above.

Our network enables us for the first time to estimate the climate response of tree growth as a function of elevation over the main dendrochronological study regions of the Europe, Asia and North America. Babst et al. [62] stated that “such work is time- and cost-intensive, but crucial for different research communities, forest management practices and for determining and implementing effective environmental policies”. We agree with this statement and need to stress that more tree-ring studies across altitudinal gradients are urgently needed to verify the conclusions presented here.

## Supporting Information

Figure S1 **Locations of the study area, tree-ring sampling site and the nearest meteorological station.**
(TIF)

Figure S2 **Local climatic conditions shown as monthly mean, maximum and minimum temperatures (left panel), precipitation and relative humidity (right panel) at the Jiuquan instrumental station over the common period 1951–2011.**
(TIF)

Figure S3 **Sample depth along the range of site elevations.**
(TIF)

Figure S4 **Correlation (color bars) and response functions (lines with circles) between climate data and the two age-dependent tree-ring residual series from previous July to current September over their common available period.** (a) Correlations with monthly temperature, (b) correlations with monthly minimum temperature, (c) correlations with monthly maximum temperature, (d) correlations with monthly precipitation, (e) correlations with monthly PDSI, (f) correlations with monthly relative humidity. The horizontal dashed lines indicate the 95% confidence level for the correlation function. Response functions significant at the p = 0.05 levels are marked with an asterisk above the corresponding bars.
(TIF)

Figure S5 **Multi-taper method (MTM) power spectra for the four altitudinal standard chronologies.**
(TIF)

Table S1 **Correlation coefficients of the daily mean temperature (left lower panel) and precipitation (right upper panel) from three automated weather station data over an altitudinal range of ∼700 m in the central Qilian Mountain (∼38°26′ N, 99°56′ E) during two consecutive years of monitoring in 2011 and 2012.**
(DOC)

Table S2 **Correlation coefficients of temperature and precipitation between our selected Jiuquan station data and CRU TS3.10 datasets, which were derived from two grid points (39.25°N, 98.25°E and 39.25°N, 98.75°E) with corresponding elevations of 3783 m and 3237 m, over the common 1951–2009 period.**
(DOC)

Table S3 **Correlation coefficients of the four residual (left lower panel) and standard (right upper panel) chronologies over the common reliable period 1560–2011.**
(DOC)

Table S4 **Correlation coefficients between the high-pass filtered data with the degrees of freedom (right upper panel) and the low-pass filtered data with the adjusted degrees of freedom (left lower panel) over the “most reliable” period 1560–2011.**
(DOC)

Appendix S1 **Statistics used to assess the characteristics of the tree-ring chronology in** **Table 1** **.**
(DOC)
