# Supplementary figures and images for: Climate Control on Tree Growth at the Upper and Lower Treelines: A Case Study in the Qilian Mountains, Tibetan Plateau
Source: PLoS One. 2013 Jul 11;8(7):e69065. doi: 10.1371/journal.pone.0069065 (PMC3708892; doi:10.1371/journal.pone.0069065)

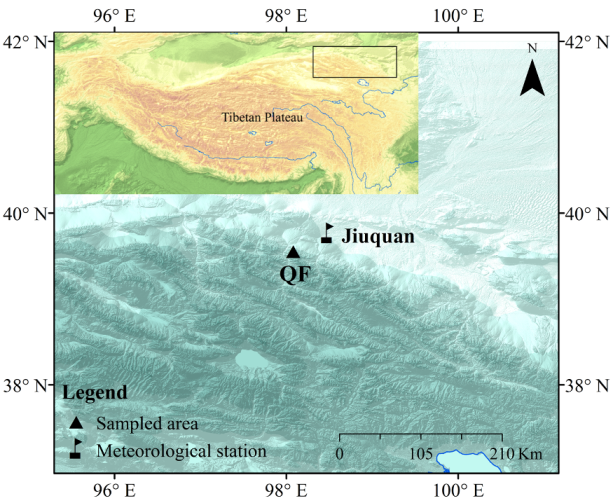

Supplement: Figure S1 — Locations of the study area, tree-ring sampling site and the nearest meteorological station. (TIF) [file pone.0069065.s001.tif]

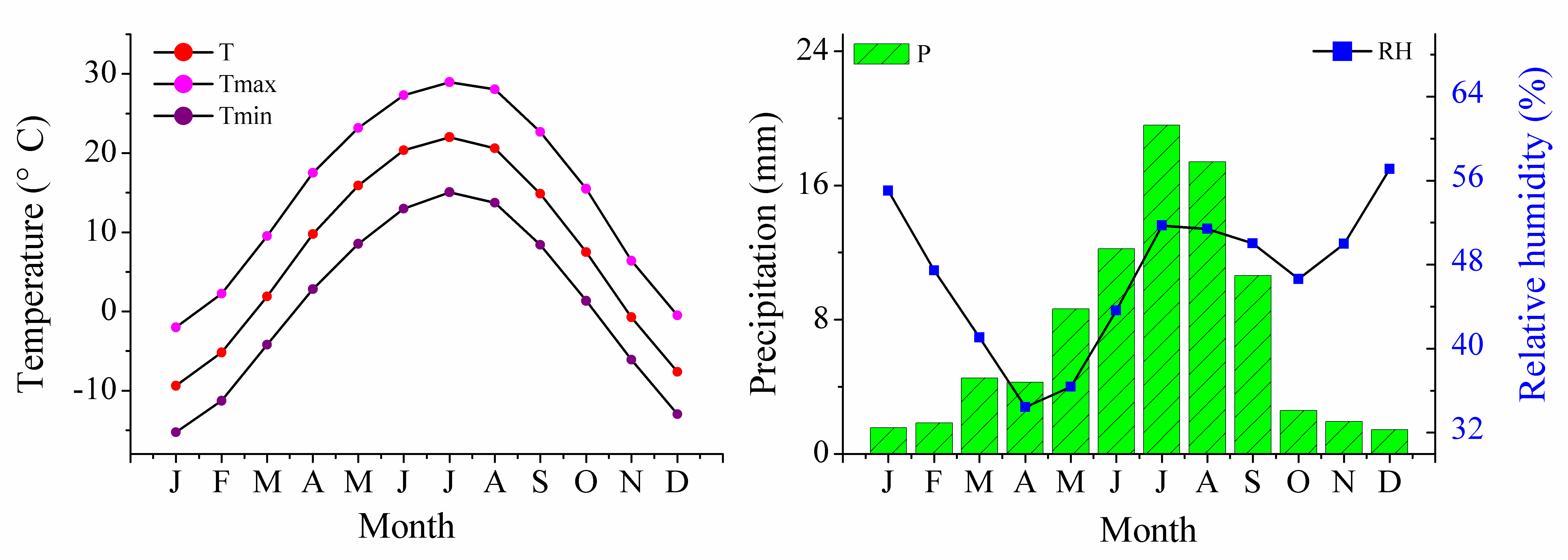

Supplement: Figure S2 — Local climatic conditions shown as monthly mean, maximum and minimum temperatures (left panel), precipitation and relative humidity (right panel) at the Jiuquan instrumental station over the common period 1951–2011. (TIF) [file pone.0069065.s002.tif]

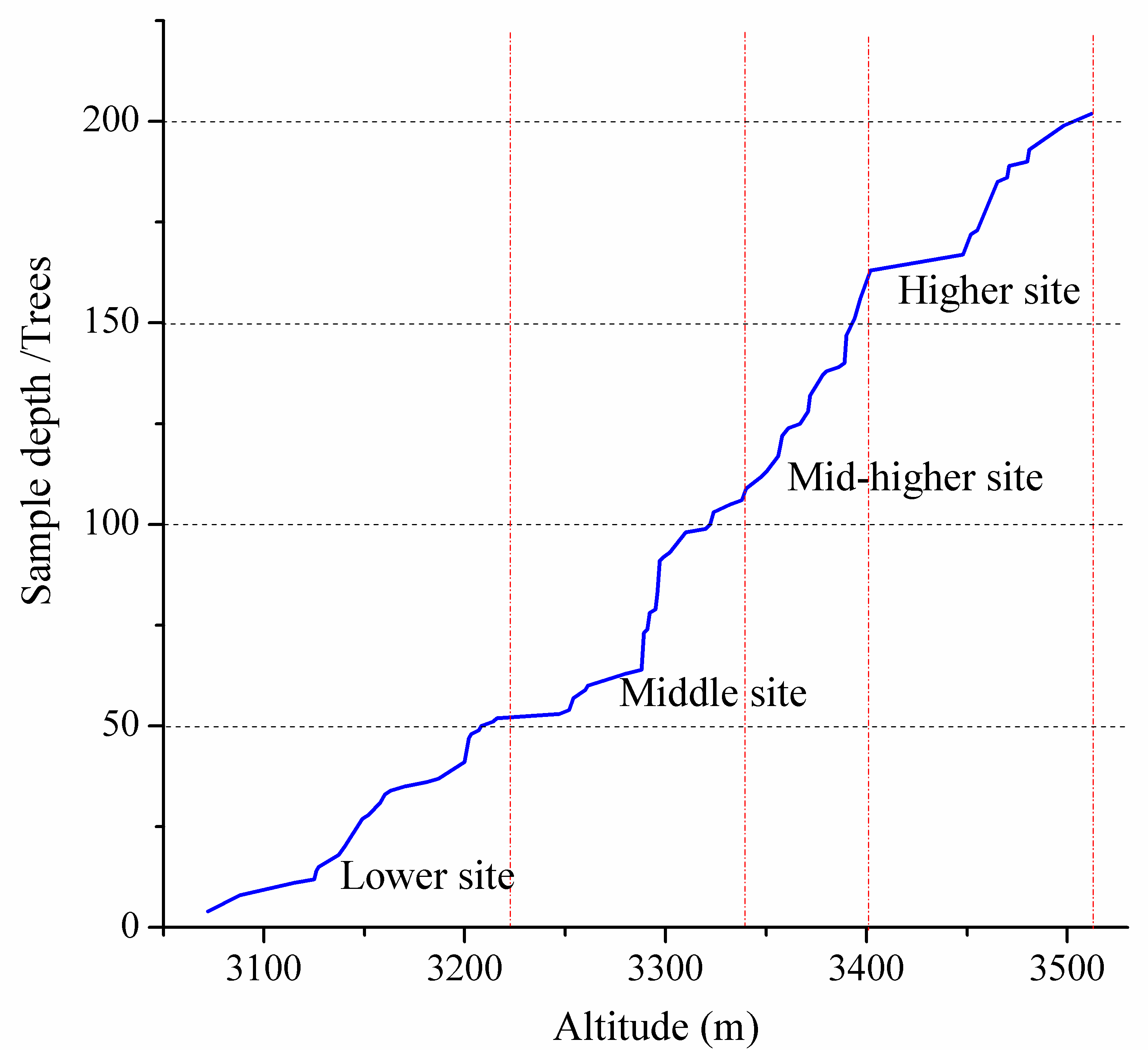

Supplement: Figure S3 — Sample depth along the range of site elevations. (TIF) [file pone.0069065.s003.tif]

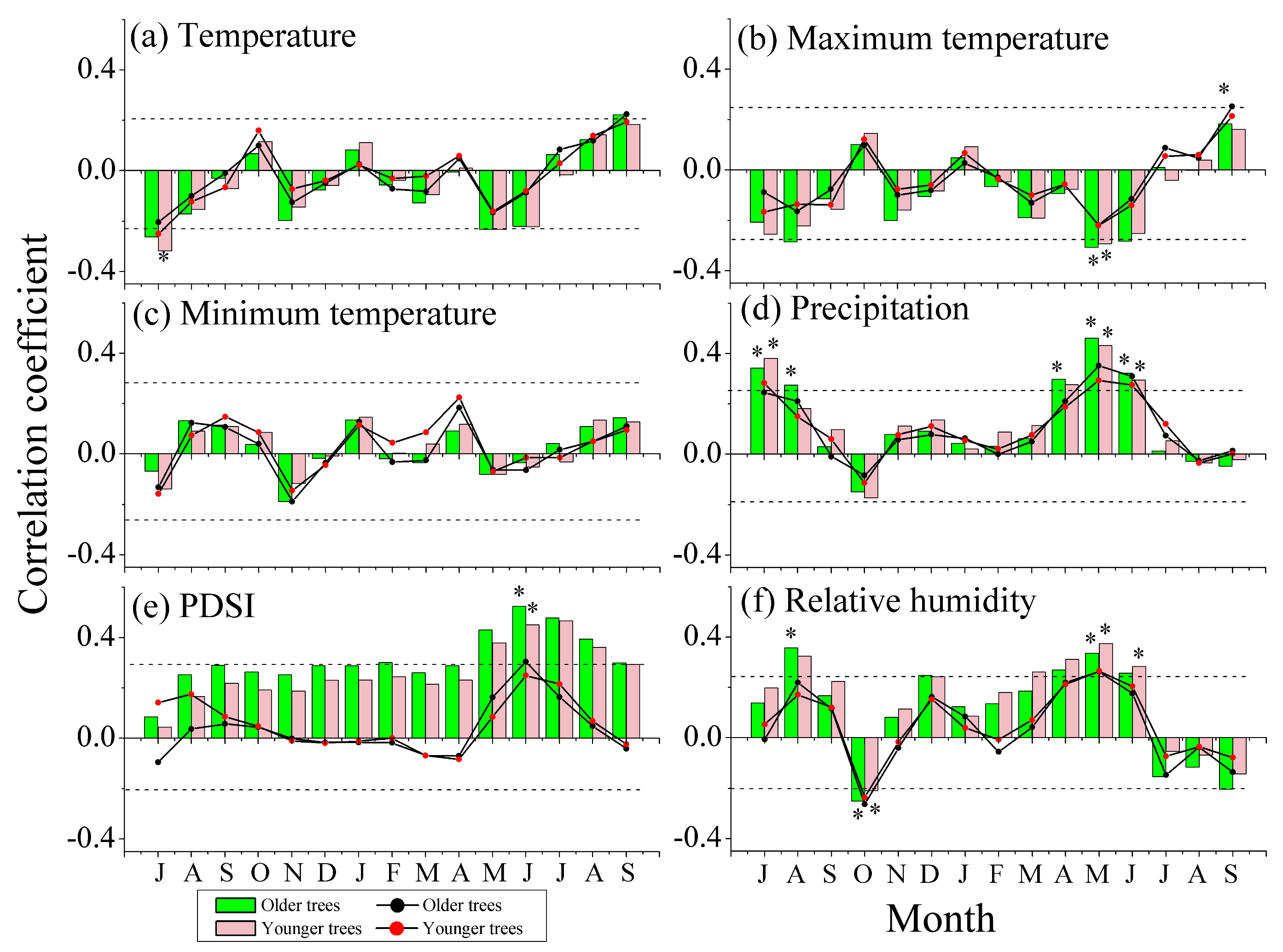

Supplement: Figure S4 — Correlation (color bars) and response functions (lines with circles) between climate data and the two age-dependent tree-ring residual series from previous July to current September over their common available period. (a) Correlations with monthly temperature, (b) correlations with monthly minimum temperature, (c) correlations with monthly maximum temperature, (d) correlations with monthly precipitation, (e) correlations with monthly PDSI, (f) correlations with monthly relative humidity. The horizontal dashed lines indicate the 95% confidence level for the correlation function. Response functions significant at the p = 0.05 levels are marked with an asterisk above the corresponding bars. (TIF) [file pone.0069065.s004.tif]

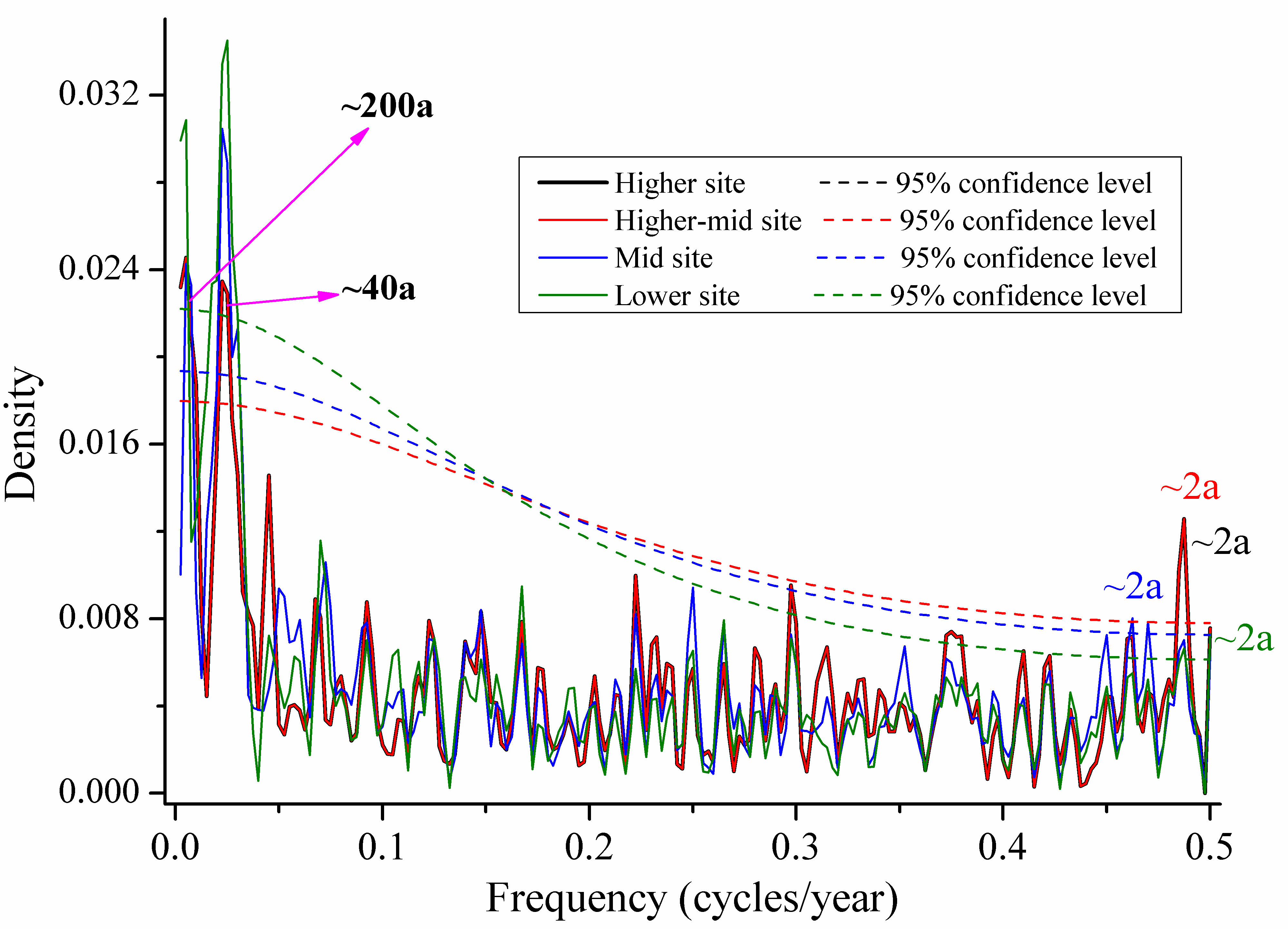

Supplement: Figure S5 — Multi-taper method (MTM) power spectra for the four altitudinal standard chronologies. (TIF) [file pone.0069065.s005.tif]
