# Supplementary material for: Climate Control on Tree Growth at the Upper and Lower Treelines: A Case Study in the Qilian Mountains, Tibetan Plateau
Source: PLoS One. 2013 Jul 11;8(7):e69065. doi: 10.1371/journal.pone.0069065 (PMC3708892; doi:10.1371/journal.pone.0069065)
Supplement: Table S1 — Correlation coefficients of the daily mean temperature (left lower panel) and precipitation (right upper panel) from three automated weather station data over an altitudinal range of ∼700 m in the central Qilian Mountain (∼38°26′ N, 99°56′ E) during two consecutive years of monitoring in 2011 and 2012. (DOC) [file pone.0069065.s006.doc]

**Table S1** Correlation coefficients of the daily mean temperature (left lower panel) and precipitation (right upper panel) from three automated weather station data over an altitudinal range of ~700 m in the central Qilian Mountain (~38°26′ N, 99°56′ E) during two consecutive years of monitoring in 2011 and 2012.

|  | UL | ML | LL |
| --- | --- | --- | --- |
| UL | 1 | 0.972** | 0.913** |
| ML | 0.996** | 1 | 0.937** |
| LL | 0.987** | 0.996** | 1 |

UL means upper limit station data (3550 m a.s.l.); ML means middle limit station data (3200 m a.s.l.); LL means lower limit station data (2865 m a.s.l.). N=688. ** denotes that correlation is significant at the p < 0.001 level.
