# Supplementary material for: Climate Control on Tree Growth at the Upper and Lower Treelines: A Case Study in the Qilian Mountains, Tibetan Plateau
Source: PLoS One. 2013 Jul 11;8(7):e69065. doi: 10.1371/journal.pone.0069065 (PMC3708892; doi:10.1371/journal.pone.0069065)
Supplement: Table S2 — Correlation coefficients of temperature and precipitation between our selected Jiuquan station data and CRU TS3.10 datasets, which were derived from two grid points (39.25°N, 98.25°E and 39.25°N, 98.75°E) with corresponding elevations of 3783 m and 3237 m, over the common 1951–2009 period. (DOC) [file pone.0069065.s007.doc]

**Table S2** Correlation coefficients of temperature and precipitation between our selected Jiuquan station data and CRU TS3.10 datasets, which were derived from two grid points (39.25°N, 98.25°E and 39.25°N, 98.75°E) with corresponding elevations of 3783 m and 3237 m, over the common 1951-2009 period.

|  | Temperature | Precipitation |
| --- | --- | --- |
| January | 0.924** | 0.861** |
| February | 0.948** | 0.648** |
| March | 0.927** | 0.959** |
| April | 0.961** | 0.924** |
| May | 0.942** | 0.939** |
| June | 0.921** | 0.757** |
| July | 0.934** | 0.753** |
| August | 0.850** | 0.939** |
| September | 0.881** | 0.951** |
| October | 0.946** | 0.963** |
| November | 0.914** | 0.851** |
| December | 0.937** | 0.881** |
| Annual mean | 0.912** | 0.834** |

** denotes that correlation is significant at the p < 0.001 level.
