# Supplementary material for: Climate Control on Tree Growth at the Upper and Lower Treelines: A Case Study in the Qilian Mountains, Tibetan Plateau
Source: PLoS One. 2013 Jul 11;8(7):e69065. doi: 10.1371/journal.pone.0069065 (PMC3708892; doi:10.1371/journal.pone.0069065)
Supplement: Table S3 — Correlation coefficients of the four residual (left lower panel) and standard (right upper panel) chronologies over the common reliable period 1560–2011. (DOC) [file pone.0069065.s008.doc]

**Table S3** Correlation coefficients of the four residual (left lower panel) and standard (right upper panel) chronologies over the common reliable period 1560-2011.

|  | Higher site | Higher-mid site | Mid site | Lower site |
| --- | --- | --- | --- | --- |
| Higher site | 1 | 0.91** | 0.87** | 0.87** |
| Higher-mid site | 0.87** | 1 | 0.92** | 0.92** |
| Mid site | 0.82** | 0.89** | 1 | 0.91** |
| Lower site | 0.80** | 0.89** | 0.88** | 1 |

** denotes that correlation is significant at the p = 0.01 level.
