# Supplementary material for: Climate Control on Tree Growth at the Upper and Lower Treelines: A Case Study in the Qilian Mountains, Tibetan Plateau
Source: PLoS One. 2013 Jul 11;8(7):e69065. doi: 10.1371/journal.pone.0069065 (PMC3708892; doi:10.1371/journal.pone.0069065)
Supplement: Table S4 — Correlation coefficients between the high-pass filtered data with the degrees of freedom (right upper panel) and the low-pass filtered data with the adjusted degrees of freedom (left lower panel) over the “most reliable” period 1560–2011. (DOC) [file pone.0069065.s009.doc]

**Table S4** Correlation coefficients between the high-pass filtered data with the degrees of freedom (right upper panel) and the low-pass filtered data with the adjusted degrees of freedom (left lower panel) over the “most reliable” period 1560-2011.

|  | HS | HMS | MS | LS |
| --- | --- | --- | --- | --- |
| HS | 1 | 0.93**/451 | 0.89**/451 | 0.88**/451 |
| HMS | 0.76**/15.4 | 1 | 0.93**/451 | 0.92**/451 |
| MS | 0.62**/17.5 | 0.81**/15.3 | 1 | 0.91**/451 |
| LS | 0.63**/16.2 | 0.83**/14.0 | 0.82**/16.1 | 1 |

HS denotes the higher-site; HMS is the higher-mid site; MS is mid-site; LS is lower-site. The mark ** denotes that correlation is significant at the p = 0.01 level.
