# Supplementary material for: Climate Control on Tree Growth at the Upper and Lower Treelines: A Case Study in the Qilian Mountains, Tibetan Plateau
Source: PLoS One. 2013 Jul 11;8(7):e69065. doi: 10.1371/journal.pone.0069065 (PMC3708892; doi:10.1371/journal.pone.0069065)
Supplement: Appendix S1 — Statistics used to assess the characteristics of the tree-ring chronology in Table 1 . (DOC) [file pone.0069065.s010.doc]

Appendix: Statistics used to assess the characteristics of the tree-ring chronology in Table 1 (all the equations are from Fritts [1]).

$SD_{x}=\sqrt{\frac{1}{n-1}\sum_{t=1}^{t=n} (x_{t}-m_{x})^{2}}$ (1)

where ∑ is the symbol for summations of the elements following it, *x_t_* is the ordered ring-width sequence such that time (indicated by subscript t) varies sequentially from *1* (the calendar year of the earliest dated ring) to *n* (the last calendar year). The symbols above and below the summation sign indicate these two limits. The mean *m_x_* is calculated by the following formula:

$m_{x}=\frac{1}{n}\sum_{t=1}^{t=n} x_{t}$ (2)

with the notation following Equation (1).

$MS_{x}=\frac{1}{n-1}\sum_{t=1}^{t=n-1} \left.\left|\frac{2(x_{t+1}-x_{t})}{x_{t+1}+x_{t}}\right.\right|$ (3)

where *x_t_* is each datum and the vertical lines designate the absolute value (neglecting the sign) of the term enclosed by them. The denominator of the term scales the absolute values of the differences between adjacent ring widths, *x_t_* and *x_t+1_*, so that the differences are proportional to the average of the two widths. The values of mean sensitivity range from 0 where there is no difference to 2 where a zero value occurs next to a nonzero value in the time sequence.

$R_{xy}=\frac{\sum_{t=1}^{t=n} (x_{t}-m_{x})(y_{t}-m_{y})}{(n-1)s_{x}s_{y}}$ (4)

where *m_x_, m_y_, s_x_, s_y_* are the means and standard deviations of the two sets of data; and *n* is the number of items compared, namely, the sample size. The correlation coefficients between the two data sets being compared can range from an upper value of +1, which indicates perfect and direct agreement, to a value of -1, which indicates perfect and inverse agreement. If the two data sets are completely independent or random with respect to one another, the correlation coefficient is zero.

Reference:

Fritts HC (1976) Tree rings and climate. Academic Press, London.
